# Supplementary material for: Retrospective Study of the Seroprevalence of HIV, HCV, and HBV in Blood Donors at a Blood Bank of Western Mexico
Source: Pathogens. 2021 Jul 11;10(7):878. doi: 10.3390/pathogens10070878 (PMC8308904; doi:10.3390/pathogens10070878)
Supplement: Supplementary file 1 [file pathogens-10-00878-s001.zip › pathogens-1255769-supplementary.pdf]

Table S1. HIV, HCV, and HBV seroreactive blood donors classified by Educational level and Occupation.

		HIV			HCV			HBV		
		**Sero-reactive**	**Rate**	**CI (95%)**	**Sero-reactive**	**Rate**	**CI (95%)**	**Sero-reactive**	**Rate**	**CI (95%)**
**Educational level**	Unlettered	1	0.12	0.00-0.37	4	0.50	0.01-0.99	1	0.12	0.00-0.37
	Elementary	11	1.37	0.56-2.18	40	4.98	3.43-6.52	17	2.11	1.11-3.12
	Junior high school	32	3.98	2.60-5.36	108	13.43	10.90-15.97	57	7.09	5.25-8.93
	High school	49	6.10	4.39-7.80	95	11.82	9.44-14.19	52	6.47	4.71-8.23
	Degree	43	5.35	3.75-6.95	91	11.32	9.00-13.64	42	5.22	3.64-6.80
	Master's degree	3	0.37	0.00-0.80	6	0.75	0.15-1.34	2	0.25	0.00-0.59
	PhD	1	0.12	0.00-0.37	2	0.25	0.00-0.59	0	0.00	does not apply
	Technical school	8	1.00	0.31-1.68	15	1.87	0.92-2.81	5	0.62	0.08-1.17
	Knows how to reed	1	0.12	0.00-0.37	1	0.12	0.00-0.37	0	0.00	does not apply
	Other	1	0.12	0.00-0.37	0	0.00	does not apply	0	0.00	does not apply
**Occupation**	Housewife	9	1.12	0.39-1.85	39	4.85	3.33-6.37	25	3.11	1.89-4.33
	Farmer	5	0.62	0.08-1.17	4	0.50	0.01-0.99	4	0.50	0.01-0.99
	Merchant	8	1.00	0.31-1.68	26	3.23	1.99-4.48	8	1.00	0.31-1.68
	Unemployed	6	0.75	0.15-1.34	6	0.75	0.15-1.34	3	0.37	0.00-0.80
	Domestic employee	0	0.00	does not apply	1	0.12	0.00-0.37	1	0.12	0.00-0.37
	Employee	88	10.95	8.66-13.23	198	24.63	21.20-28.06	89	11.07	8.77-13.37
	Student	7	0.87	0.23-1.52	17	2.11	1.11-3.12	20	2.49	1.40-3.58
	Retired	1	0.12	0.00-0.37	4	0.50	0.01-0.99	0	0.00	does not apply
	Laborer	5	0.62	0.08-1.17	11	1.37	0.56-2.18	8	1.00	0.31-1.68
	Professional	2	0.25	0.00-0.59	11	1.37	0.56-2.18	7	0.87	0.23-1.52
	Construction worker	4	0.50	0.01-0.99	2	0.25	0.00-0.59	1	0.12	0.00-0.37

Rates are expressed in cases per 10,000 and age range in years. HIV = Human Immunodeficiency Virus; HCV = Hepatitis C Virus; HBV= Hepatitis B Virus; CI = Confidence intervals (95%).
